# Supplementary figures and images for: Genetic and environmental influences on the distributions of three chromosomal drive haplotypes in maize
Source: PLoS Genet. 2025 Jul 16;21(7):e1011742. doi: 10.1371/journal.pgen.1011742 (PMC12279129; doi:10.1371/journal.pgen.1011742)

A

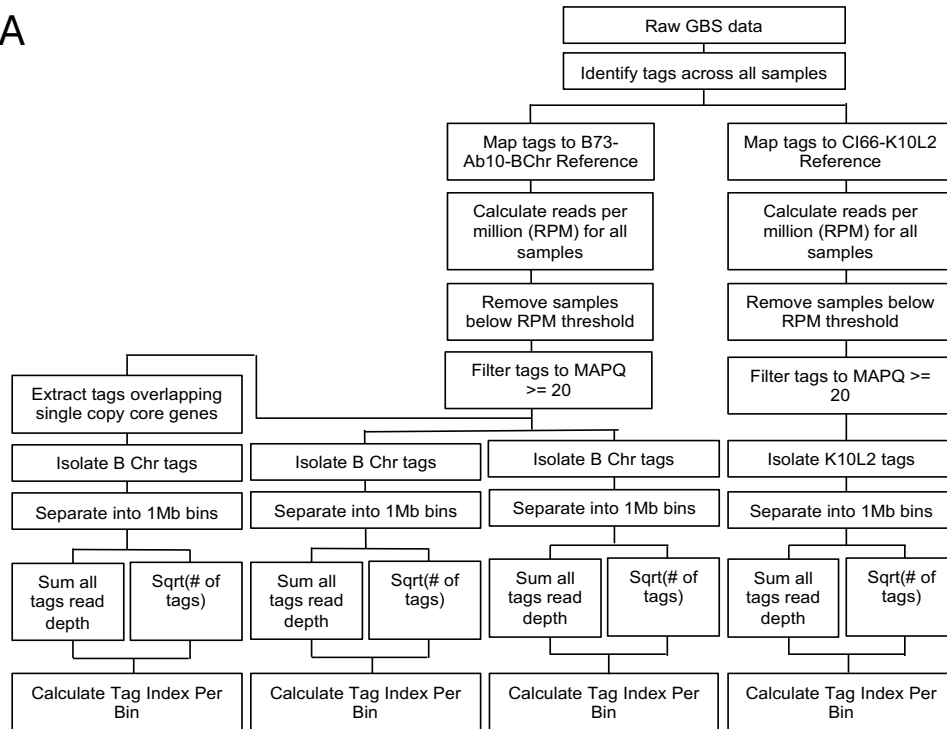

B

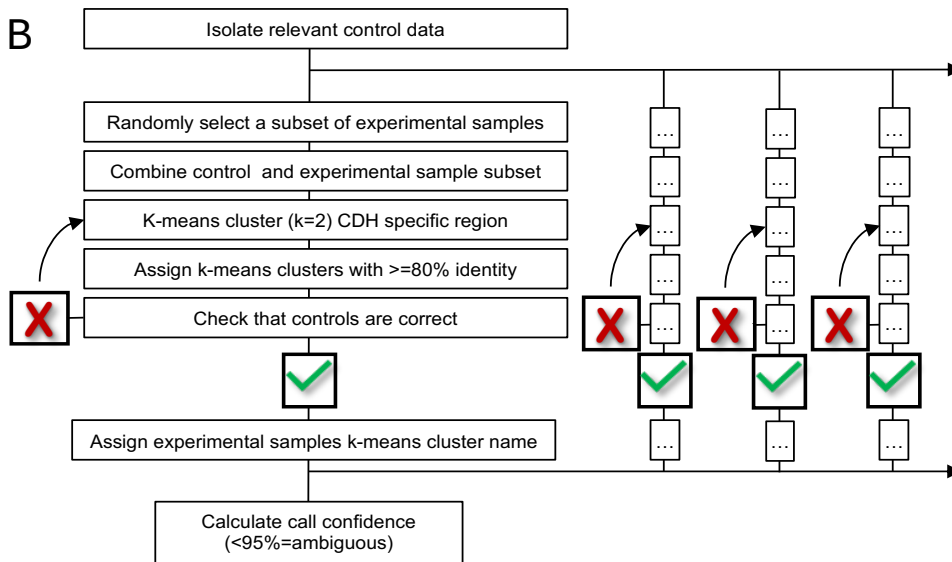

Supplement: S1 Fig — Workflow diagrams. A. Workflow diagram for the generation of the tag index for CDHs and single copy core genes. In brief, we first identified tags, which are identical 64 bp sequences. We then mapped those tags to the relevant reference genome. Then we calculated reads per million for each mapped tag and filtered to remove unreliable tags. To calculate tag index, we isolated the CDH and divided it into 1 Mb bins. On each bin we calculated the tag index. B. Diagram of the workflow for detecting CDHs in experimental samples. Check indicates passing, x indicates failing. (PDF) [file pgen.1011742.s006.pdf]

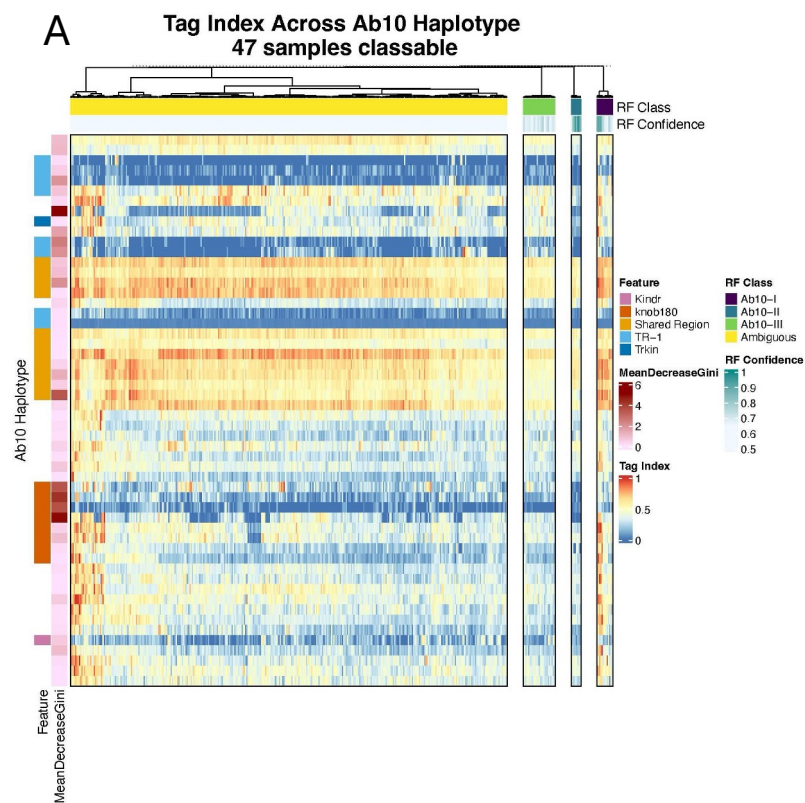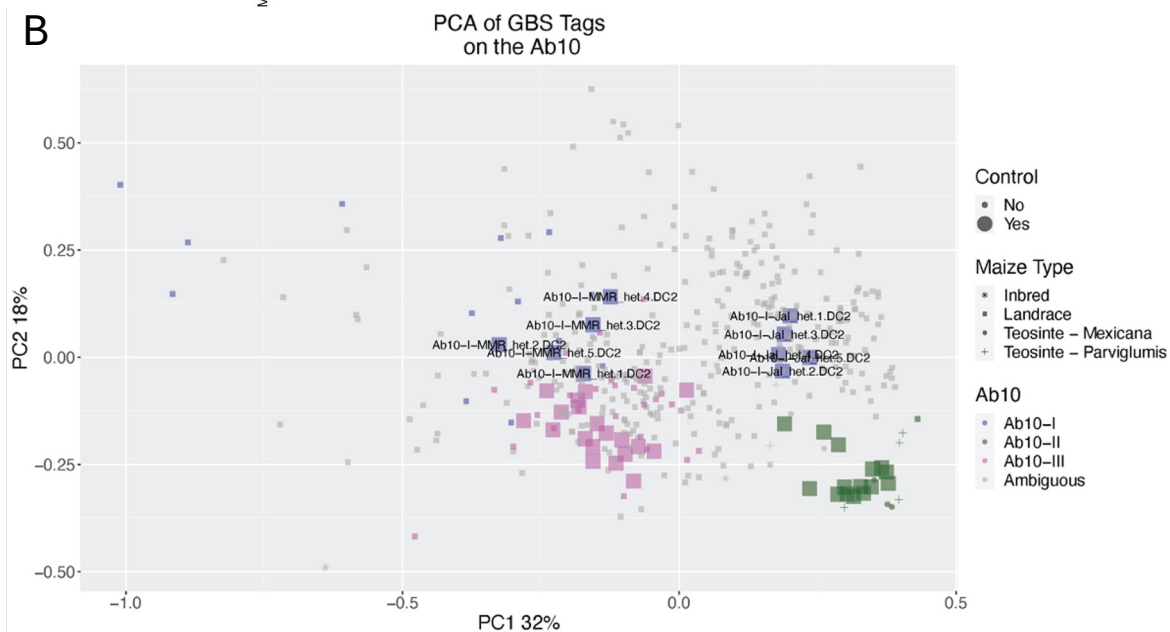

Supplement: S2 Fig — Identification of Ab10 type. A. Results of a random forest model trained on control data and applied to all experimental Ab10 positive samples. Ab10 types classified by the random forest model are plotted separately and each group is ward.D clustered. The x axis shows individual samples, the y axis shows features of the Ab10 haplotype and the importance of each 1 Mb bin in determining Ab10 type in the random forest model (Mean Decrease Gini). The RF confidence value indicates the proportion of decision trees that are called the predominant Ab10 type. B. A PCA of all the Ab10 positive samples scaled tag index with controls and their type indicated. Control samples are shown in large shapes colored by their Ab10 class, experimental samples are shown in small shapes. Experimental samples that were confidently classed by the random forest model are shown in their respective color. (PDF) [file pgen.1011742.s007.pdf]

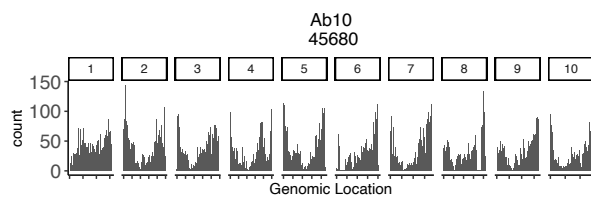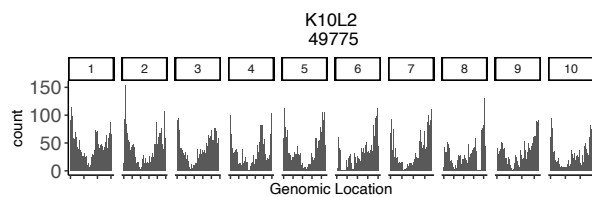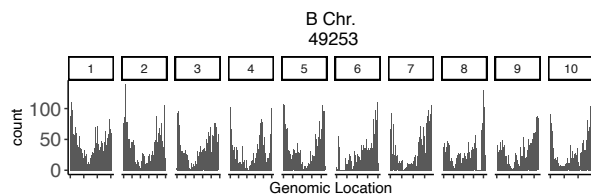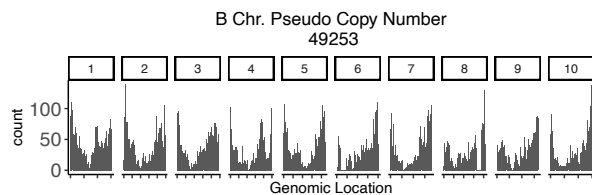

Supplement: S3 Fig — Location of SNPs used for GWAS. Numbers below the CDH name indicate the total number of SNPs. (PDF) [file pgen.1011742.s008.pdf]

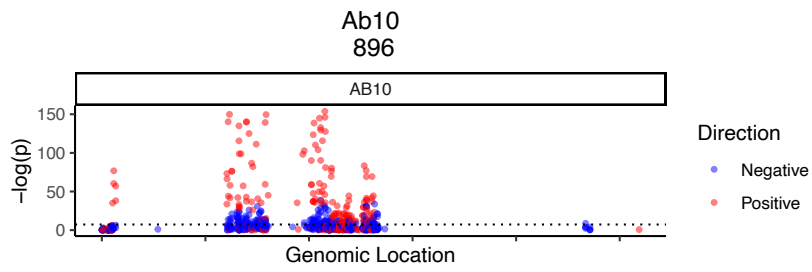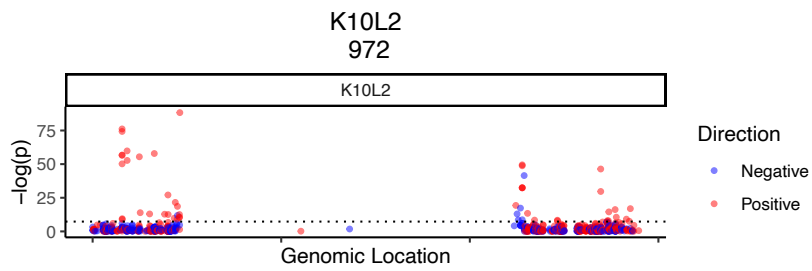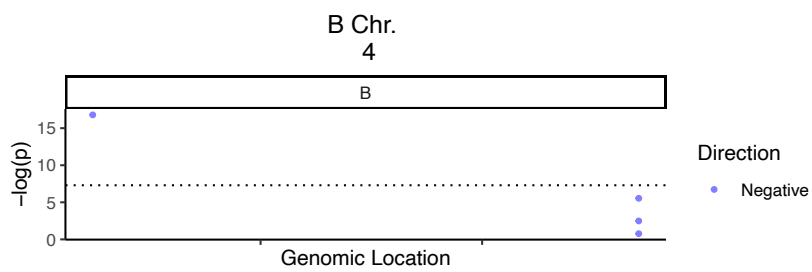

Supplement: S4 Fig — Manhattan plots of SNPs that passed through filtering but lie within or close to a CDH. Numbers below the CDH names indicate the number of SNPs in the plot. Dotted grey line indicates a p value of 5x10^-8^. Negatively associated loci are the result of alleles linked to the non-CDH homologs (the N10 shared regions). (PDF) [file pgen.1011742.s009.pdf]

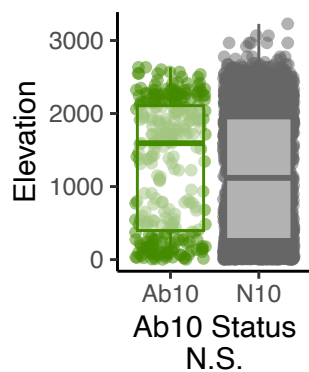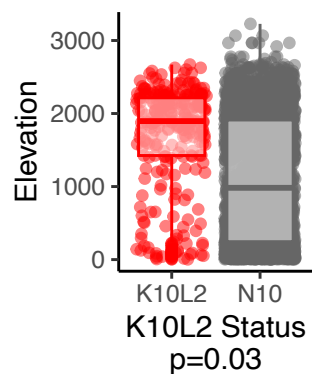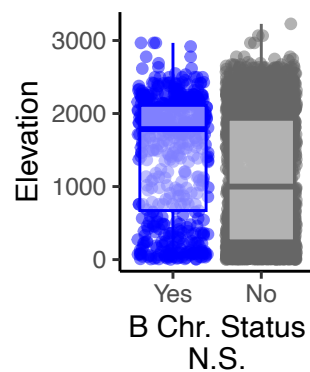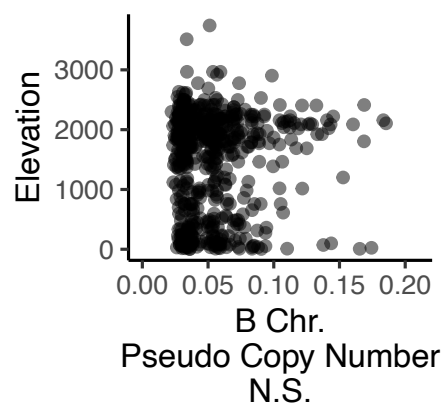

Supplement: S5 Fig — Relationship of CDHs to elevation. Each point represents a sample either with or without a CDH and the elevation at which they were originally collected. The Ab10, K10L2, and the B Chr. data were analyzed using a generalized linear model with a binomial family and the following model: CDH presence/absence ~ all 10 whole genome SNP principal components + elevation of collection. For B chromosome copy number the same formula was used with a linear family. N.S. indicates not significant. (PDF) [file pgen.1011742.s010.pdf]

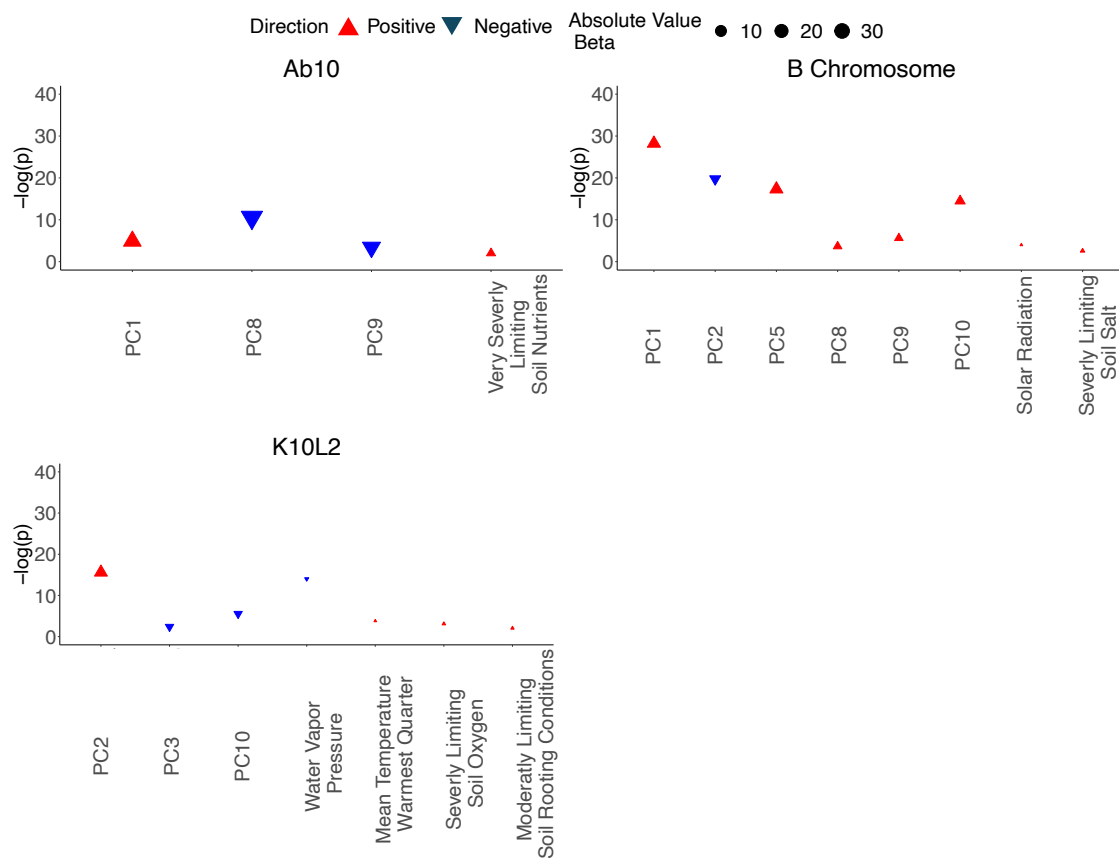

Supplement: S6 Fig — Plots of simplified generalized linear models for each CDH including population structure and environmental variables (but not genetic modifiers). Shape color and orientation indicate the direction of the relationship to the CDH. Shape size represents the effect size. (PDF) [file pgen.1011742.s011.pdf]

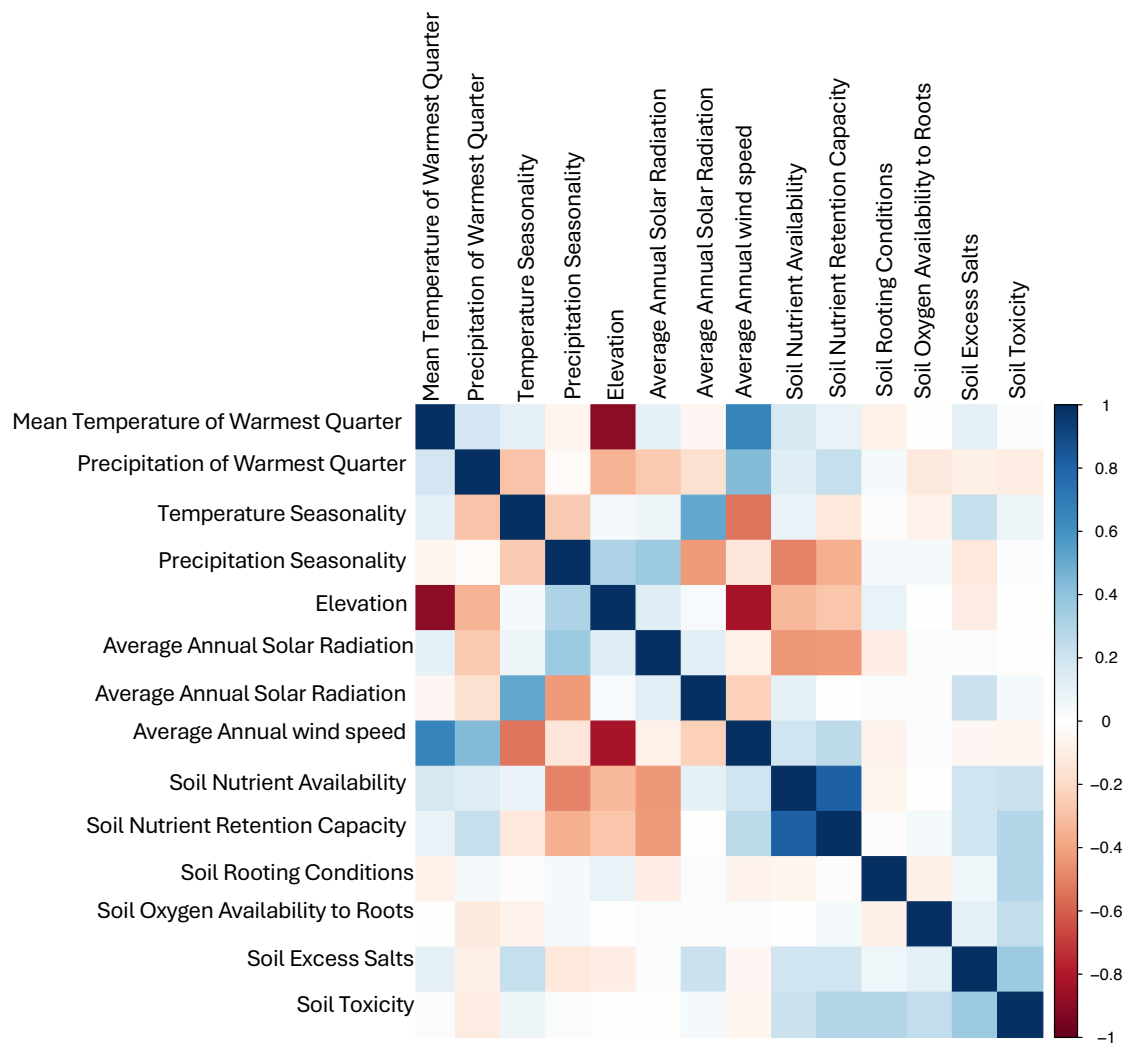

Supplement: S7 Fig — Correlation matrix for selected environmental variables. (PDF) [file pgen.1011742.s012.pdf]
